# Long-term (≥ 15 years) outcome quality after Class II:1 bionator or Herbst multibracket appliance treatment: A comparison

**DOI:** 10.1007/s00056-023-00457-3

**Published:** 2023-02-24

**Authors:** Niko Christian Bock, Rebecca Jungbauer, Ingrid Rudzki, Peter Proff, Sabine Ruf

**Affiliations:** 1 Department of Orthodontics, Justus Liebig University, Giessen, Germany; 2 Department of Orthodontics, University Medical Centre Regensburg, Franz-Josef-Strauß-Allee 11, 93053 Regensburg, Germany; 3 Faculty of Medicine, Ludwig Maximilian University, Munich, Germany

**Keywords:** Long-term stability, Functional treatment, Peer Assessment Rating index, Overjet, Occlusal relationship, Langzeitstabilität, Funktionelle kieferorthopädische Apparaturen, Peer Assessment Rating index, Overjet, Okklusion

## Abstract

**Purpose:**

To compare the long-term outcome quality (≥ 15 years) of Class II:1 treatment using either a bionator (BIO) or a Herbst–multibracket appliance (HMB).

**Methods:**

Patients who underwent functional treatment during the ideal treatment period for the respective approach (prepuberty vs. peak/postpeak) were assessed. Inclusion criteria were overjet ≥ 4 mm, skeletal Class II and availability of study casts from before, after and ≥ 15 years after treatment. The study casts were assessed using the Peer Assessment Rating (PAR) index and standard orthodontic cast measurements.

**Results:**

During treatment, PAR score, overjet and sagittal occlusal relationship improved significantly in all groups. Long-term, there was a significant increase of incisor irregularity in the upper (HMB) and lower (BIO) arch and a significant decrease of lower arch width 3 – 3 (BIO). PAR score, overjet, and sagittal occlusal relationship remained stable long-term. Intergroup comparisons revealed significant differences between the BIO and HMB groups in terms of lower arch width (6 – 6), upper and lower arch width (3 + 3/3 – 3) as well as sagittal molar relationship.

**Conclusions:**

The achieved improvement in PAR score, overjet, and sagittal occlusion remained comparably stable long-term in all groups. The long-term changes are probably a consequence of natural aging.

**Supplementary Information:**

The online version of this article (10.1007/s00056-023-00457-3) contains supplementary material, which is available to authorized users.

## Abstract (de)

**Ziel:**

Der Vergleich der langfristigen Ergebnisqualität (≥ 15 Jahre) einer Klasse-II:1-Behandlung mit einem Bionator (BIO) oder einer Herbst-Multibracket-Apparatur (HMB).

**Methoden:**

Patienten, die während des idealen Behandlungszeitraums für den jeweiligen Therapieansatz (vor der Pubertät bzw. Peak/Post-Peak) funktionskieferorthopädisch behandelt worden waren. Die Einschlusskriterien waren: Overjet ≥ 4 mm, skelettale Klasse II, Studienmodelle von vor, nach und ≥ 15 Jahre nach der Behandlung vorhanden. Die Studienmodelle wurden anhand des Peer Assessment Rating (PAR) index und kieferorthopädischer Standardmessungen ausgewertet.

**Ergebnisse:**

Während der Behandlung verbesserten sich PAR-Score, Overjet und sagittale Okklusionsbeziehung in allen Gruppen signifikant. Langfristig kam es zu einer signifikanten Zunahme des Engstandes der Schneidezähne im oberen (HMB) bzw. unteren (BIO) Zahnbogen und zu einer signifikanten Abnahme der Breite des unteren Zahnbogens 3 – 3 (BIO). PAR-Score, Overjet und sagittale Molarenrelation blieben langfristig stabil. Die Intergruppenvergleiche ergaben signifikante Unterschiede zwischen der BIO- und der HMB-Gruppe in Bezug auf die untere Zahnbogenbreite (6 – 6), die obere und untere Zahnbogenbreite (3 + 3/3 – 3) sowie die sagittale Molarenbeziehung.

**Schlussfolgerungen:**

Die erzielte Verbesserung des PAR-Scores, des Overjets und der sagittalen Molarenrelation blieb in allen Gruppen langfristig vergleichbar stabil. Die langfristigen Veränderungen sind wahrscheinlich eine Folge physiologischer Alterungsprozesse.

## Introduction

Class II:1 malocclusion is known to have a prevalence of 8.1–16.2% [32, 66, 67]. There are several therapeutic approaches that are sometimes discussed controversially; one of them is functional orthopaedics. Basically, functional appliances can be divided into two main groups: removable and fixed appliances.

Removable functional appliances have a very long history dating back into the 19th century [41]. Over the years, variable appliance designs have been described such as the activator [1] and bionator appliance [7] and their further developments and modifications, e.g. the Balter’s bionator in the modification by Ascher [36, 38]. Most of these appliances are indicated predominantly in growing patients [10, 22] presenting a Class II:1 with a favourable facial morphology and favourable inherited growth potential of the mandible [34, 36].

The original basic idea behind the classical functional appliance is to eliminate all habits and parafunctions as early as possible so that the setting of the final inherited neutral (Class I) occlusion is not endangered during puberty. So, with respect to the literature, the ideal treatment (Tx) period for this specific functional therapeutic approach is prepuberty, in order to enable maximal mandibular growth [5, 39, 44, 68].

Fixed functional appliances were introduced slightly later [31] but their routine use started only a few decades ago [40, 48, 50, 53]. These appliances are mainly used in the permanent dentition and after the peak of pubertal growth. The effects of the Herbst appliance have been shown to be a combination of skeletal and dental changes in both the maxilla and the mandible [49], with the immediate Tx results being independent of the growth pattern [6, 11, 52, 53, 58, 59, 62], but without changing the inherited vertical growth pattern long-term [20, 52, 58].

For both removal and fixed functional appliances, controversial opinions exist in terms of their effectiveness and the stability of Tx results [17, 19, 42, 70]. While a certain amount of data exist on the immediate Tx effects and short-term stability [19, 42, 70], data regarding the long-term effects during adulthood are scarce for both Tx options. This applies also for comparisons between different appliances regarding the respective post-Tx changes and long-term outcome quality.

## Aim

Therefore, the aim of the present investigation was to compare the long-term outcome quality in patients who underwent functional (removable vs. fixed) Class II Tx during the ideal Tx period recommended for the corresponding Tx approach (prepuberty vs. at peak/postpeak of the pubertal growth).

## Subjects and methods

Orthodontic study casts and lateral cephalograms of patients who were treated with

**Fig. 1:**
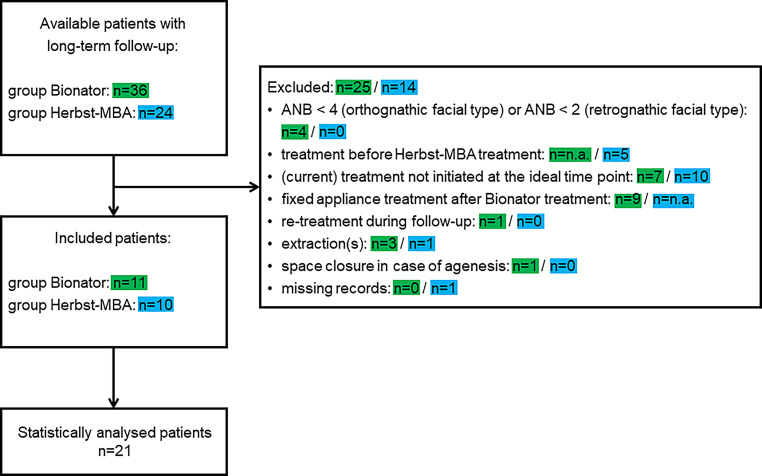
Flowchart exhibiting the effect of the exclusion criteria on the two original patient samples. *MBA* multibracket appliance, *n.a.* not applicable Flussdiagramm, das die Auswirkung der Ausschlusskriterien auf die beiden ursprünglichen Patientengruppen zeigt. *MBA* Multibracket-Apparatur, *n.a.* nicht zutreffend

- A Balter’s bionator modified by Ascher [5] only, prepuberty (group BIO) and
- A Herbst appliance followed by a multibracket appliance peak/postpeak (group HMB) were retrospectively collected from the Departments of Orthodontics, University Medical Centre Munich and University of Giessen, both Germany. To be included in the investigation patients had to meet the following inclusion criteria (Fig. 1):
  - Class II:1 with an overjet ≥ 4 mm before Tx,
  - Skeletal Class II (ANB > 2° in retrognathic/4° in orthognathic/6° in prognathic facial types [63]),
  - Class II Tx according to one of the protocols given above performed during the ideal skeletal maturity period recommended for the respective Tx approach,
  - Hand–wrist radiographs from before Tx (T0) available,
  - Lateral cephalograms from before Tx (T0) available,
  - Study casts from before Tx (T0), after treatment (T1) and ≥ 15 years after treatment (T2) available and
  - No agenesis of permanent teeth, extraction therapy or space opening/closure, no craniofacial syndromes.

**Fig. 2:**
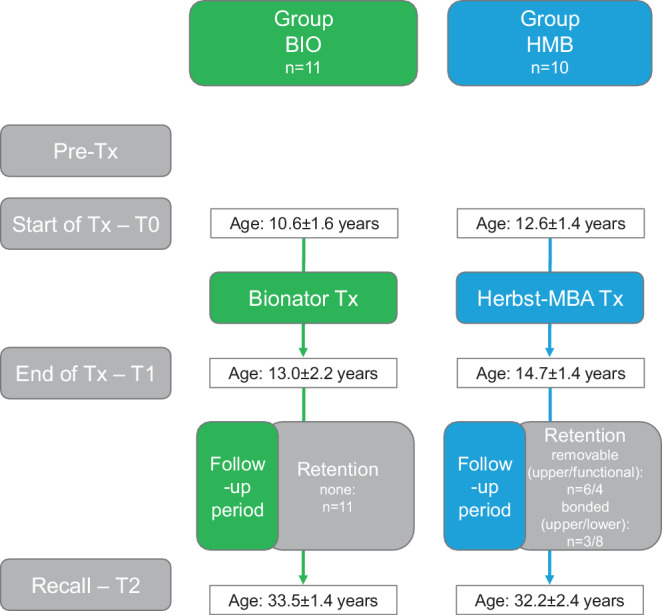
Flowchart describing the observation time points and periods of the two groups. *BIO* bionator, *HMB* Herbst–multibracket appliance (MBA), *Tx* treatment Flussdiagramm zur Beschreibung der Beobachtungszeitpunkte und -zeiträume der beiden Gruppen. *BIO* Bionator, *HMB* Herbst–Multibracket-Apparatur (MBA), *Tx* Therapie

All subjects participated in a long-term follow-up examination at one of the two departments of orthodontics: the group BIO at the University of Munich and the HMB group at the University of Giessen. Ethical approval was obtained separately at the two locations (Munich 77/97 and Giessen 146/13). All patients gave written informed consent. A brief overview of the Tx protocols is given in Fig. 2, while detailed descriptions can be found elsewhere for the BIO group [38, 57] and the HMB group [12].

### Methods

Existing study models from all time points (T0, T1, T2) were assessed. The peer assessment rating (PAR) index [55, 56] was used in order to generate objective data on outcome quality; all ratings were performed by two certified/calibrated assessors according to the respective guidelines. In addition, the following variables were assessed:

- Sagittal molar relationship (right, left) in cusp widths (cw),
- Sagittal canine relationship (right, left) in cusp widths (cw),
- Overjet in mm,
- Overbite in mm,
- Mandibular and maxillary incisor irregularity measured according to Little’s irregularity index [45],
- Upper arch perimeter in mm,
- Lower arch perimeter in mm,
- Upper arch width (molar, canine) in mm and
- Lower arch width (molar, canine) in mm.

Visual ratings of the sagittal molar and canine relationships were performed to the nearest 0.25 cusp widths (cw) and classified as Class I, II or III. Linear measurements on dental casts were made using a digital calliper (HSL 246-15, Hammacher, Solingen, Germany) with a precision of 0.01 mm. Prior to the statistical evaluation all measurements were rounded to the first decimal.

### Measurement error

To minimize the error of the method, all assessments were performed twice—once each by N. B. and R. J.; the mean value of both measurements was used as the final measurement value.

The method error (ME) was calculated (a) using the formula of Dahlberg [18]:

$ME=\sqrt{\frac{\sum d^{2}}{2n}}$, where d is the difference between two registrations and *n* is the sample size and (b) using the intraclass correlation coefficient (ICC, two-way mixed, absolute agreement) to determine interrater reliability. The data are shown in the supplementary Table 1.

### Statistical assessment

Due to the explorative character of the study, no sample size calculation was performed. The arithmetic mean (M), standard deviation (SD), and median (MD) as well as interquartile range (IQR) were calculated for each variable. The data were tested regarding normal distribution using the Shapiro–Wilk test and visual assessment of the histograms showing a nonnormal distribution in more than 5%. Therefore, nonparametric Mann–Whitney U tests were applied for intergroup comparison long-term and Friedman tests followed by post hoc pairwise comparison for longitudinal changes within groups. For the post hoc test the Bonferroni* p*-value correction was applied. The following levels of significance were utilized: *p* < 0.001, *p* < 0.01 and *p* < 0.05; *p* ≥ 0.05 was considered as not significant (n. s.). In case the global test indicated significant changes, but the post hoc test yielded *p*-values *p* ≥ 0.05 due to the Bonferroni correction, the results of the global test were rated nonsignificant. The effect size (r) was calculated and interpreted in accordance with Cohen: 0.1 = small, 0.3 = medium 0.5 = large [16].

## Results

**Fig. 3:**
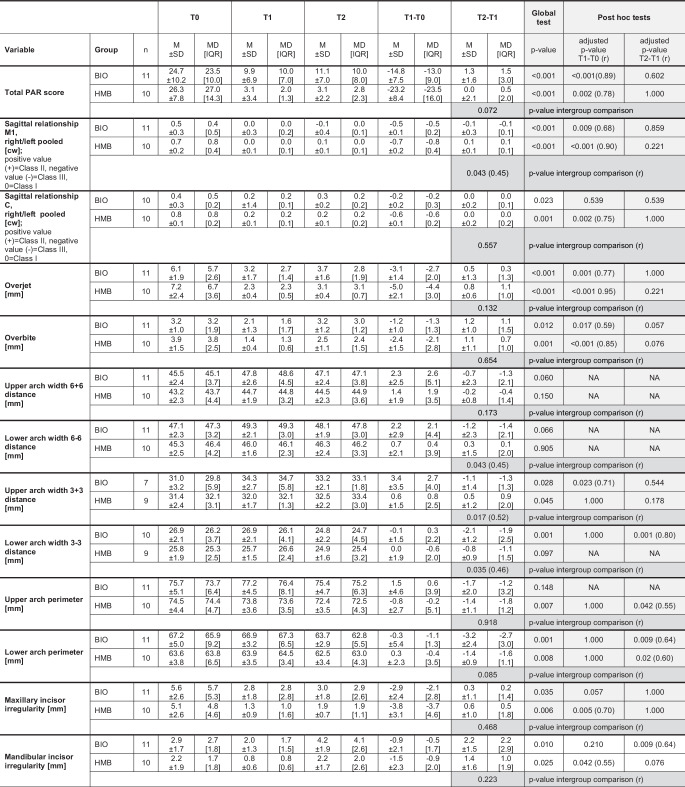
Descriptive and analytical outcome of the intra- and intergroup comparison during the different time periods. Global changes were tested with nonparametric Friedman’s two-way analysis of variance by ranks for intragroup changes (rows) followed by post hoc pairwise comparisons and with Mann–Whitney U tests for intergroup comparison long-term (columns), r is the effect size (< 0.3 small effect, 0.3–0.5 medium effect, > 0.5 large effect). *T0* before treatment, *T1* after treatment, *T2* ≥ 15 years after treatment, *n* numbers analysed, *M* mean, *SD* standard deviation, *MD* median, *IQR* interquartile range, *M1* first molars, *C* canines, *cw* cusp widths, *NA* not applicable Deskriptive und analytische Ergebnisse des Intra- und Intergruppenvergleichs über die verschiedenen Zeiträume. Globale Veränderungen wurden mit nichtparametrischen Friedman-Tests hinsichtlich gruppeninterner Veränderungen (Zeilen), gefolgt von paarweisen Post-Hoc-Vergleichen und mit Mann-Whitney-U-Tests für den Langzeitvergleich zwischen den Gruppen (Spalten) getestet, r ist die Effektstärke (< 0,3 schwacher Effekt, 0,3–0,5 mittlerer Effekt, > 0,5 starker Effekt). *T0* vor der Behandlung, *T1* nach der Behandlung, *T2* ≥ 15 Jahre nach der Behandlung, *n* Anzahl der analysierten Patienten, *M* Mittelwert, *SD* Standardabweichung, *MD* Median, *IQR* Interquartilsbereich, *M1* erste Molaren, *C* Eckzähne, *cw* Prämolarenbreiten, *NA* nicht zutreffend

Based on the inclusion criteria a total of 21 patients could be included (Fig. 1): BIO group: 11 (6 female/5 male), HMB group: 10 (7 female/3 male). Their baseline characteristics are presented in Fig. 3.

### Intragroup comparison

#### Active Tx (T1–T0)

In both groups a significant reduction of the mean PAR score (14.8–23.2), overjet (3.1–5.0 mm) and overbite (1.2–2.4 mm) occurred. According to the PAR score categorisation, the ratio greatly improved/improved/worse or no different at T1 was 18%/73%/9% in the BIO group and 70%/30%/0% in the HMB group. Sagittal molar and canine relationships were significantly improved (0.2–0.7 cw), although sagittal canine relationship improvement in the BIO group was not significant. In the HMB group a significant reduction of incisor irregularity in both dental arches (maxilla: 3.8 mm; mandible: 1.5 mm) could be detected, whereas the corresponding reduction in the group BIO (maxilla: 2.9; mandible: 0.9 mm) was not significant (Fig. 3). The group BIO, however, was the only one to show a significant increase of upper arch width 3 + 3 distance.

#### Long-term follow-up (T2–T1)

None of the mean post-Tx changes of the variables PAR score (0.0–1.3), sagittal molar and canine relationships (−0.1 to 0.1 cw) or overjet (0.5–0.8 mm) were significant in any of the groups (Fig. 3). At T2, the PAR score categorisation (greatly improved/improved/worse or no different) revealed a ratio of 18%/82%/0% in the BIO and 50%/50%/0% in the HMB group.

The HMB group exhibited a significant average decrease in upper arch perimeter (1.4 mm), even though the decrease was similar in the BIO group (1.7 mm). All groups showed a significant decrease of the mean lower arch perimeter (1.4–3.2 mm). In the BIO group the mean lower arch width (3 – 3 distance) also decreased significantly (2.1 mm). A significant increase in mean mandibular incisor irregularity was seen in the BIO (2.2 mm) group (Fig. 3).

### Intergroup comparison

#### Long-term follow-up (T2–T1)

For the majority of variables, no significant group difference was obvious. This, however, was not true for upper and lower arch width 3 + 3/3 – 3 distance, where a mean decrease of 1.1/2.1 mm was seen in the BIO group, while an increase of 0.5/decrease of 0.8 mm occurred in the HMB group; and the lower arch width 6 – 6 exhibited a decrease in the BIO (1.2 mm) and an increase in the HMB group (0.3 mm). The sagittal molar relationship showed also a significant difference with a further decrease towards a slight Class III occlusion (0.1 cw) in the BIO and a slight increase towards Class II occlusion (0.1 cw; Fig. 3).

### Measurement error

For the PAR index the interrater reliability was good or excellent (0.8–1). The method error calculated by using Dahlberg’s formula ranged from 0.1 to 1.74. The detailed results are presented in supplementary Table 1.

## Discussion

The present retrospective investigation is the first to compare long-term (≥ 15 years) outcome and stability after functional Class II Tx using different approaches. For natural reasons, the two groups differed in terms of age and skeletal maturity, as Tx was mandatory to have taken place during the ideal skeletal maturity period recommended for the respective Tx approach (prepuberty and peak/postpeak, respectively). Consequently, intergroup comparison was only performed for the long-term interval. Due to this inclusion criterion and the necessity of study casts from ≥ 15 years after Tx, the sample size of the individual groups was rather small. Nevertheless, the Tx approach was identical within each of the groups. The investigation is based on study casts which were evaluated by two examiners. For most variables the interrater reliability can be considered as very good.

Looking at the results in terms of outcome quality (total PAR score), both groups showed significant improvement during Tx (T0–T1). Nevertheless, the post-Tx PAR score remains distinctly higher in the BIO group than in the HMB group. This difference can be explained by the fact that the patients in the BIO group did not undergo additional multibracket appliance Tx for finishing after functional appliance therapy. Franscisconi et al. reported a mean reduction of the PAR score by 24.7 ± 6.6 as a result of Tx with a bionator and a multibracket appliance [25], which is a similar reduction as in the HMB group. Nevertheless, both groups showed only small mean changes of total the PAR score (range 0.0–1.3) during the long-term post-Tx observation period (T2–T1), which is in accordance with previous studies [12, 25, 38].

In terms of the sagittal and vertical occlusal variables both groups showed similar post-Tx values (T1). On average, the molar relationship was Class I and the canine relationship was up to 0.25 cw Class II which can still be considered normal [2].

Post-Tx mean overjet and overbite values were within a “physiological” range as well (2.3–3.2 mm and 1.4–2.1 mm, respectively). All parameters (molar relationship, overjet and overbite) showed rather good stability during the long-term post-Tx observation period (T1–T2) with the final values being similar as in untreated subjects without orthodontic Tx need during adolescence [12, 29]. Although there was a significant group difference long-term considering molar relationship with minimal tendency towards Class II occlusion in the HMB group (+0.1 cw) and towards Class III occlusion (−0.1 cw) in the BIO group, the amount of the change is not relevant from a clinical perspective. In the BIO group, only patients with favourable inherited growth potential of the mandible were included, which might explain the tendency towards a Class III occlusion which naturally takes place during the residual growth phase. Thus, in terms of stability of the achieved occlusal outcome it does not seem to make a difference long-term whether to treat early during the mixed dentition using a removable functional appliance or to do so at a later stage in the permanent dentition with a fixed functional appliance.

Looking at the transverse dimension, for upper and lower arch width 6 ± 6 distance none of the groups exhibited a significant change, neither during Tx nor during the long-term observation period. The bionator group was the only group that was not treated with any kind of fixed appliances and the gain in the posterior transverse distance is very likely only due to development and natural growth [46]. The minor, nonsignificant, changes during the long-term interval are in accordance with the findings by Bondevik et al. [13]. Nevertheless, there was a significant group difference long-term for the lower intermolar width (6 – 6) with a decrease only in the BIO group. Henrikson et al. reported a significantly greater decrease of lower intermolar width in male compared to female between the age of 13.6 and 31.1 years [30]. In the present study the proportional relation female/male in the BIO group was 6/5, whereas in the HMB group 7/3, which might be the reason for the greater decrease in the BIO compared to the HMB group. Furthermore, the BIO group was without any appliance or retention for a longer time interval compared to the HMB group (20.5 and 17.5 years).

For upper arch width 3 + 3 distance, however, a significant increase occurred during T1–T0 in the BIO group, probably due to natural growth and development of the dental arches as they were treated prepuberty [46, 47]. And this variable showed a significant difference between the groups BIO and HMB (T2–T1) with a decrease only in the BIO group. In the HMB group, however, 3 patients had bonded upper retainers and in addition all patients wore either an upper removable or a functional appliance, whereas none of the BIO patients wore any appliance for retention after the end of Tx. Regarding lower arch width 3 – 3, a significant change (decrease) was only determined for the BIO group during the long-term observation period. It is well known from literature that lower arch width 3 – 3 remains stable after the eruption of the permanent canines during the growth period and decreases over the years which is very likely related to aging processes [13, 15, 21, 46, 64]. In contrast to the BIO group most of the subjects in the Herbst group had been given a bonded cuspid retainer at T1, which is probably the reason for the significant long-term group difference. Nevertheless, a reduction of the 3 – 3 distance also occurred in the Herbst group. It remains unclear whether the reduction of lower arch width 3 – 3 in the HMB group was caused by failures of the retainers, removal of the bonded retainers or unwanted tooth movement in spite of retainers.

Looking at the lower arch perimeter, a significant mean decrease (range 1.4–3.5 mm) occurred in both groups during the long-term observation period. In the HMB group, however, the amount was much smaller, which might be explained by the fact that—in comparison to the other group—many subjects (60%) still wore a bonded retainer at the time of the recall. Regarding the upper arch perimeter, an average decrease of 1.4–1.7 mm was determined in both groups, but was significant in the HMB group only. The physiological age dependent reduction of both the upper and lower arch perimeters long-term is in accordance with the literature [9, 15, 28, 69].

Maxillary and mandibular incisor irregularity showed a significant mean decrease during T0–T1 in the HMB group. It can be assumed that due to no fixed appliance Tx, changes in the bionator group the reduction was less pronounced. Nevertheless, especially the incisor irregularity in the upper arch improved, which might be owing to the gain of space due to the significant increase in the upper arch with 3 + 3. During the long-term observation, a significant mean increase occurred in the BIO group. The group HMB showed slightly less increase, which might also be attributed to the fact that the majority of subjects in this group still had a bonded retainer in place at the time of the recall. Again, the increase of incisor irregularity in the mandible related to aging is well known [13, 15, 51, 64] and is very likely associated with the decrease in arch perimeter and intercanine width [69]. But of course, it might also be kind of a side effect related to incisor proclination during active Tx—particularly in the HMB group; on the other hand, no such long-term effect was seen when investigating the effects of Herbst Tx on the mandibular incisor segment [27].

In the literature, it is often recommended to treat patients with functional appliances during pubertal growth [3, 8, 23, 33, 54]. The original therapeutic approach of removable Class II functional appliances, however, was less to directly stimulate mandibular growth but primarily to enable the full development of the inherited pubertal growth potential by eliminating all habits and parafunctions as early as possible, namely in the prepuberty phase [4, 5, 7, 35, 37, 39]. Therefore, for the present investigation, the ideal period for bionator Tx was considered as prepuberty. Compliance with removable functional appliances can be limited resulting in prolonged Tx or even impeding unsuccessful Tx [14, 65], but seems to be substantially better during prepuberty [24, 43]. In contrast, fixed functional appliances are mainly used during later stages of dental and skeletal development, respectively. According to the literature the ideal period for Herbst Tx is in the permanent dentition at or just after the peak of pubertal growth [61]. Even if mandibular growth stimulation has been shown to be possible even in young adults [60], the largest amount of sagittal condylar growth was demonstrated in subjects treated around the peak of the pubertal growth period [26].

Retrospective long-term follow-up studies have several limitations to consider. As it is very often impossible to locate and include all patients and not every potential patient agrees to take part in the follow-up, this kind of study is inherently biased but also the number of subjects very limited. Furthermore, patients that were satisfied with the former Tx might be more likely to be willing to participate as those that were not satisfied with the Tx result. The different groups were treated by different orthodontists, which might be considered as further possible bias. Due to the retrospective design and limited sample size there is of course a limited power and generalizability; in addition, no untreated control group with similar long-term data was available. Another limitation is that long-term cephalometric images were not available for ethical reasons. Therefore, only dental parameters could be evaluated, which surely limits the validity of the Tx effectiveness within the Tx groups.

The aim of this study was to evaluate and compare the long-term stability of the different Tx approaches and not the comparison their Tx effectiveness, nor to investigate their equivalence. BIO or HMB treatments are indicated at different stages of growth and therefore are not comparable or interchangeable. For each patient, an individual decision must be made as to which treatment approach is best, depending on the skeletal, not the dental, morphology, and growth stage, as well as compliance.

Although the results of the present study need to be interpreted with caution, the data are unique.

## Conclusion

Both functional treatment approaches (bionator treatment, Herbst–multibracket appliance treatment) resulted in substantial reduction in Peer Assessment Rating (PAR) score, overjet and sagittal occlusion. Long-term, these treatment results exhibited good stability in all groups. Long-term changes that have occurred in the dentition are most likely a consequence of the aging process.

### Supplementary Information

Supplementary Table 1
