# Supplementary material for: Long-term (≥ 15 years) outcome quality after Class II:1 bionator or Herbst multibracket appliance treatment: A comparison
Source: J Orofac Orthop. 2023 Feb 24;85(4):260–9. doi: 10.1007/s00056-023-00457-3 (PMC11186930; doi:10.1007/s00056-023-00457-3)
Supplement: Supplementary file 1 — Supplementary Table 1 [file 56_2023_457_MOESM1_ESM.pdf]

**Supplementary Table 1:** Evaluation of systematic and casual error of measurements

**Ergänzende Tabelle 1:** Bewertung des systematischen und zufälligen Messfehlers

| Variable | T0 | | T1 | | T2 | |
|---|---|---|---|---|---|---|
| | **ME** | **ICC** | **ME** | **ICC** | **ME** | **ICC** |
| **SNA [°]** | 0.7 | 0.982 | - | - | - | - |
| **SNB [°]** | 0.6 | 0.980 | - | - | - | - |
| **ANB [°]** | 0.5 | 0.939 | - | - | - | - |
| **NL/NSL [°]** | 1.0 | 0.955 | - | - | - | - |
| **ML/NSL [°]** | 1.4 | 0.965 | - | - | - | - |
| **ArGo'Gn [°]** | 1.7 | 0.965 | - | - | - | - |
| **PAR Index** | 2.0 | 0.972 | 2.0 | 0.941 | 2.1 | 0.935 |
| **Sagittal relationship M1, right side [cw]** | 0.0 | 0.982 | 0.1 | 0.801 | 0.0 | 0.991 |
| **Sagittal relationship M1, left side [cw]** | 0.1 | 0.954 | 0.1 | 0.949 | 0.1 | 0.947 |
| **Sagittal relationship C, right side [cw]** | 0.1 | 1.000 | 0.1 | 0.843 | 0.1 | 0.961 |
| **Sagittal relationship C, left side [cw]** | 0.1 | 1.000 | 0.1 | 0.851 | 0.1 | 0.922 |
| **Overjet [mm]** | 0.4 | 0.960 | 0.4 | 0.947 | 0.4 | 0.948 |
| **Overbite [mm]** | 0.7 | 0.912 | 0.4 | 0.916 | 0.4 | 0.950 |
| **Upper arch width 6-6 [mm]** | 0.5 | 0.990 | 0.4 | 0.989 | 0.6 | 0.979 |
| **Lower arch width 6-6 [mm]** | 0.7 | 0.979 | 0.6 | 0.970 | 0.6 | 0.976 |
| **Upper arch width 3-3 [mm]** | 1.3 | 0.791 | 1.5 | 0.806 | 1.5 | 0.791 |
| **Lower arch width 3-3 [mm]** | 0.3 | 0.987 | 0.5 | 0.943 | 0.5 | 0.946 |
| **Upper arch perimeter [mm]** | 0.9 | 0.979 | 0.6 | 0.989 | 0.5 | 0.990 |
| **Lower arch perimeter [mm]** | 0.9 | 0.977 | 0.5 | 0.987 | 1.0 | 0.936 |
| **Maxillary incisor irregularity [mm]** | 0.8 | 0.942 | 0.6 | 0.908 | 0.6 | 0.911 |
| **Mandibular incisor irregularity [mm]** | 0.6 | 0.927 | 0.4 | 0.926 | 0.5 | 0.969 |

ME: measurement error calculated according to Dahlberg, ICC: intraclass correlation coefficient (two-way mixed, absolute agreement)

ME: Messfehler berechnet nach Dahlberg, ICC: Intraklassen-Korrelationskoeffizient (zweiseitig gemischt, absolute Übereinstimmung)
